# Recognising ‘high-risk’ eyes before cataract surgery

**Published:** 2008-03

**Authors:** Parikshit Gogate, Mark Wood

**Affiliations:** Head, Department of Paediatric Ophthalmology, Community Eye Care, HV Desai Eye Hospital, Pune 411028, India. Email: parikshitgogate@hotmail.com; Consultant Ophthalmologist, CCBRT Hospital, Box 23310, Dar es Salaam, Tanzania. Email: markwood@cats-net.com

**Figure:**
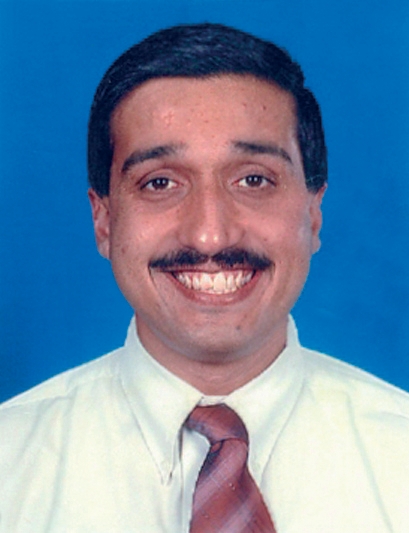


**Figure:**
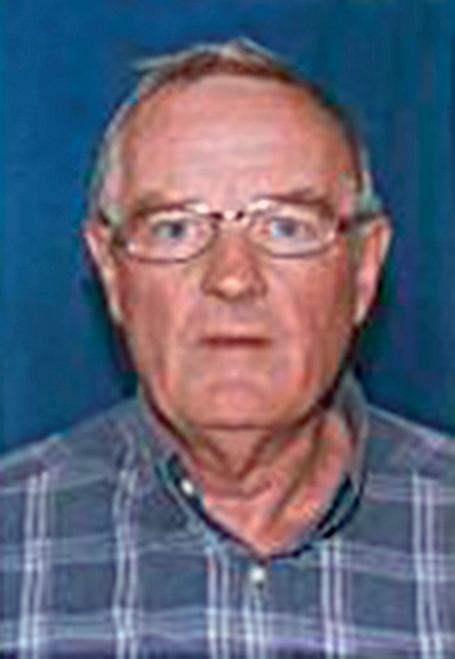


Certain eyes are at a higher risk of complication during cataract surgery. Operations on such ‘high-risk’ eyes are also more likely to yield a poor visual outcome (defined as best corrected vision less than 6/60 after surgery).^1^

Learning to recognise when eyes are at greater risk, and acting accordingly, will help you to avoid complications. Even so, before the operation takes place, it is good practice to explain to such patients that a poor outcome is a possibility. This makes these patients' expectations more realistic and improves postoperative compliance and follow-up. In most cases, patients who are blind with complicated cataract will be happy with even a modest improvement of their vision.

It is also important to have available all the equipment you may need to manage a possible complication, for example a vitrectomy machine in the case of capsular rupture and vitreous loss.

Depending on where you are in the world, certain ‘high-risk’ eyes will be more common: for example, pseudoexfoliation in Somalia and India, onchocerciasis in Sudan, and angle-closure glaucoma in Asia. You will get to know your local problems as you perform more operations.

## Before you operate

Get an accurate patient history. In particular, obtain information on trauma, previous operations, diabetes, dry eye, amblyopia, and congenital abnormalities. If the patient has only one eye, it is necessary to find out what caused the loss of the other eye.

Perform a thorough eye examination. This should include:

- **Measuring best corrected visual acuity.** This will determine whether a potentially risky operation should be attempted or avoided. If the patient only has one eye, is the patient content with his or her present vision? Be aware that you could make it worse.
- **A slit lamp examination with dilated pupil.** Many potential problems become visible when the pupil is dilated. A slit lamp examination will identify most problems you are liable to face during surgery, such as subluxated lenses. Check the maturity of the lens, the condition of the capsule, and whether the cataract really is the cause of the patient's poor vision, before deciding to perform a potentially risky operation.
- **Measuring intraocular pressure.** It is important to measure intraocular pressure in all patients, for example to identify glaucoma.
- **A fundus examination.** The fundus can be seen through all but the densest cataracts. You can do a B-scan if the medium is not clear.

**Figure:**
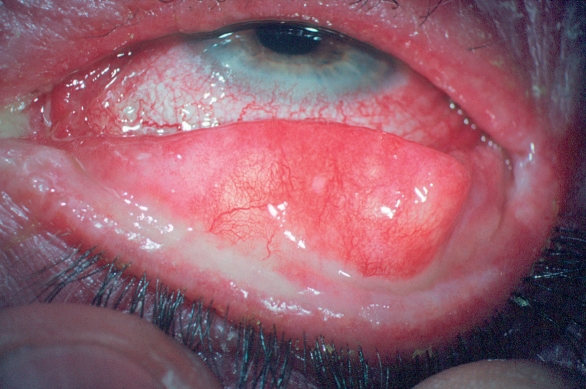
**Conjunctivitis**

Your examination should be able to identify problems or signs which indicate that the operation may not be straightforward.

During the examination, you will need to pay attention to the following areas, which are discussed in this article:

- infection
- potential visualisation problems
- anatomy of the anterior segment
- crystalline lens profiles
- other health conditions.

## Infection

Any infection in or around the eye could lead to **endophthalmitis**; infections should therefore be treated before surgery.

**A blocked and infected lacrimal sac** may cause endophthalmitis. It is extremely important to check the sac patency before surgery. If the sac has mucoid regurgitation, instil local antibiotic drops and postpone surgery. A dacryocystectomy (DCT) or dacryocystorhinostomy (DCR) may be done if antibiotics do not resolve the condition before surgery is to take place.

**Entropion, ectropion, and lagophthalmos:** these eyes may have corneal exposure before and after surgery. Eyelashes rubbing on the eye are a source of infection. In such eyes, the postoperative use of steroids may precipitate a corneal ulcer. In addition, lack of a proper lid closure mechanism will not allow the eye drops instilled to stay in the conjunctival sac. These three conditions need to be corrected by surgery before you can contemplate a cataract extraction.

**Conjunctivitis** should be treated with topical antibiotics prior to intraocular surgery.

## Potential visualisation problems during surgery

### Corneal opacity

Leucoma-grade opacity will make your task extremely difficult. You will find it difficult to see details, in particular the capsulotomy. There may be residual lens matter remaining in the bag, which will be difficult to see. It will also be challenging to place the intraocular lens (IOL) in the posterior chamber with both haptics under the iris.

Patients suffering from trachoma with pannus, corneal dystrophy, degeneration, and band-shaped keratopathy, have hazy corneas. Raised intraocular pressure may cause epithelial oedema. Phenylephrine dilating drops, if used too frequently, may cause epithelial haze. Even minimal corneal handling during surgery may decrease corneal clarity.

Older patients, and those with Fuchs' dystrophy, uveitis, or glaucoma, may have a compromised endothelium; their corneas may decompensate after surgery. The use of high-viscosity viscoelastics, such as Healon GV (sodium hyaluronate), and minimal anterior chamber manipulations may help preserve the endothelium.^2^^,^^3^ It may be advisable to perform extracapsular cataract extraction (ECCE), rather than phacoemulsification or manual small incision cataract surgery (SICS).^4^

If there is a central corneal scar obscuring the pupil, an optical sector iridectomy may be helpful.

### A small pupil

A small, rigid pupil poses a problem in both ECCE and SICS. Any unnecessary manipulation of the iris can result in a small pupil. This will make it difficult to see residual lens matter, the position of the IOL, and the anterior capsule for capsulotomy.

**Figure:**
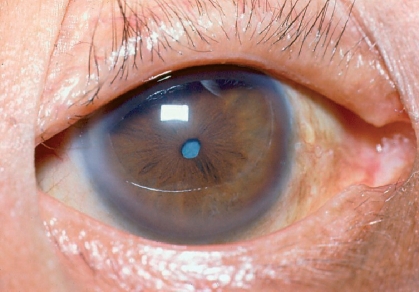
**Small pupil**

A small pupil can be dealt with in the following ways:

- Intracameral phenylephrine can be tried first.
- A Sinskey hook or Y-shaped IOL dialler can be used to stretch the pupillary sphincter. The instruments are placed 180° apart and the pupil stretched right out to the limbus for ten seconds.
- If the pupil is still too small, a sphincter-ectomy (three small radial cuts on the sphincter pupillae, 120° apart) can be done to facilitate nucleus delivery (Figure 1).
  **Figure 1:**
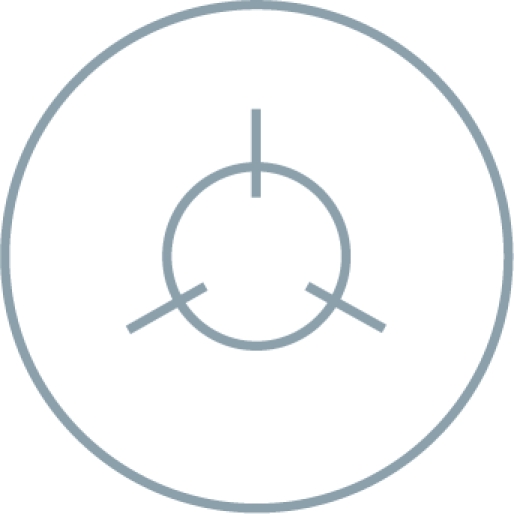
***Sphincterotomy for a small, rigid pupil: three cuts made 120° apart***
- Finally, iris hooks may be used to dilate the pupil.

**Figure 2:**
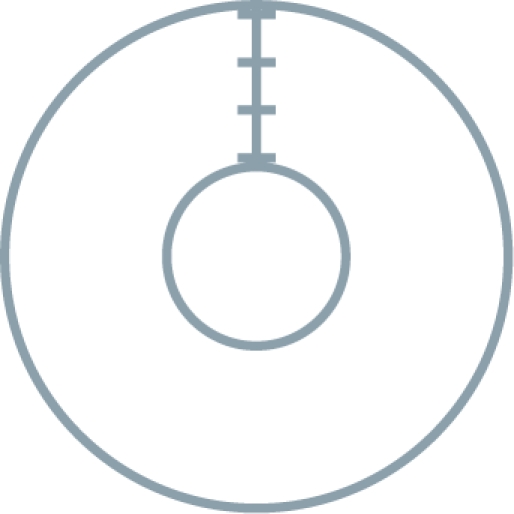
***Radial iridotomy sutured with 10.0 interrupted sutures***

If the surgeon is fastidious about having a round pupil postoperatively, a small peripheral iridectomy can be made and the cut extended to the pupillary margin (radial iridotomy). The iridotomy can be sutured later using 10-0 Prolene interrupted sutures (Figure 2); this procedure demands considerable skill and patience. However, this is not often required.

## Anatomy of the anterior segment

### Narrow anterior chambers

These eyes make it difficult to perform intraocular manipulations and to move the instruments in and out of the eye. This increases chances of iris injury and iridodialysis at the iris root (the thinnest part of the iris) and at its major arterial circle. A narrow chamber occurs in hypermetropic eyes, while deep anterior chambers occur in high myopes.

### High ametropia

High hypermetropia or myopia create specific problems.

The surgeon must re-check the A-scan and keratometer findings of IOL power, as errors are common in high myopia and high hypermetropia. It is worth trying to do a refraction to help assess the A-scan readings. Very dense and mature cataracts can give erroneous readings. When in doubt, it is better to veer on the side of slight myopia postoperatively. Most patients prefer to see clearly in the medium-to-near distance without spectacle correction.

Highly myopic patients have a wide angle, which facilitates instrument entry. However, the nucleus can be very large and the chamber deep. A deep anterior chamber may be caused by a ‘reverse pupil block’. In this situation, the iris should be raised from the capsule to even out the pressure; the chamber depth will then return to normal.^5^

Viscoelastics help to maintain anterior chamber depth where necessary and to ease the insertion of instruments. The entry and exit of instruments should be kept to a minimum.

## Crystalline lens profiles

The cataracts mentioned below will test a surgeon's skill, experience, and patience. In ECCE, a capsular tension ring (CTR) can be inserted after doing a continuous (complete) circular capsulo rhexis (CCC) to stabilise the bag.^6^ Note that it is more important that the CCC be ‘complete’ than it be ‘circular’, because an intact capsular margin ensures that the zonular tension is equally divided all around. Keep hydrodissection to a minimum. If you are using phacoemulsification, do it ‘in the bag’. If you are using ECCE or SICS, gently rotate the nucleus into the anterior chamber (do not tumble) and then deliver it outside the incision. All are difficult procedures. It may be easier to remove the lens (possible intracapsular extraction with vectis loop or lensectomy) and implant an anterior chamber lens.

- **Hard, dense nuclei** are difficult to remove with phacoemulsification or SICS. You may prefer to do a routine extracapsular extraction.^4^
- **Hypermature cataracts** have a small nucleus and a wrinkled capsule. Anterior capsulotomy may be difficult.
- **Milky cataract (Morganian):** when making the capsulotomy, the ‘milk’ from the cataract fills the anterior chamber, obscuring the surgeon's view. The anterior capsulotomy may not be complete. Filling the anterior chamber with viscoelastic before starting the capsulotomy may help.
- **Fibrotic anterior capsule:** these very thick, tough capsules may have to be cut with scissors.
- **Pseudoexfoliation** causes weak zonules and glaucoma. There is an increased chance of zonular dialysis.
- A **subluxated or dislocated lens** can occur in many conditions: very mature lenses, pseudoexfoliation, trauma, Marfan's syndrome, and other syndromes.

**Figure:**
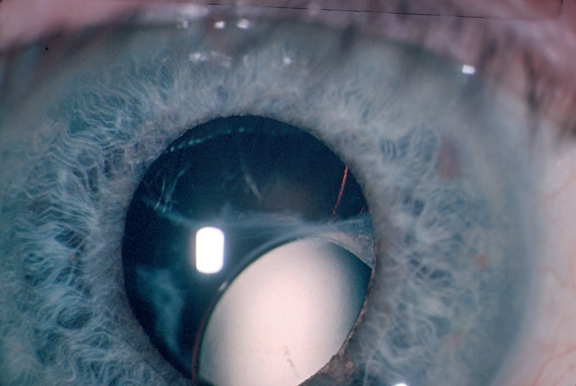
**Dislocated lens after trauma**

**Traumatic cataracts** can give nasty surprises. The following steps may help you deal with them:

- Do an ultrasound B-scan before surgery.
- There may be corneal or iris tears that need to be repaired. Make a small incision at the 12 o'clock position and use air or viscoelastic to form the anterior chamber before suturing the cornea.
- The anterior capsule may be broken or torn. The tear can be extended as a CCC or an ‘envelope’ capsulotomy.
- Keep the hydrodissection minimal, as there may be a posterior capsular tear.
- If there is a posterior capsular tear, perform dry aspiration under cover of viscoelastic.
- Anterior vitrectomy is necessary if the posterior capsule is torn. Try and preserve as much of the capsule as possible. We normally do not put an IOL in at this stage, but rather do this as a secondary procedure.

**Membranous cataract** occurs when the lens matter has been absorbed and the anterior and posterior capsules fuse. A capsulotomy, possibly followed by an anterior vitrectomy, should clear the opacity. Leave enough capsule to support an IOL. This IOL will have to be placed in the sulcus.

**Figure:**
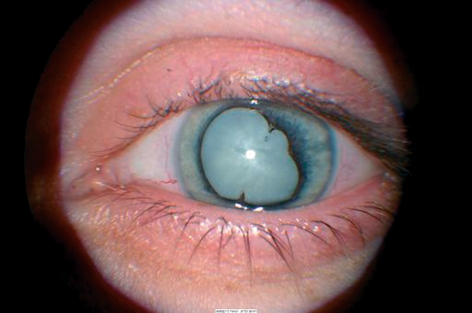
**Uveitis**

**Uveitis** causes synechiae and cataract. Posterior synechiae can be gently separated using an iris repositor after instilling viscoelastic. This will probably mean that you will perform a ‘can-opener’ capsulotomy. The sphincter pupillae may still need to be stretched. Keep iris handling to a minimum in cases of uveitis, at it may trigger postoperative inflammation. It is advisable to start oral and local steroids a few days before surgery.

## Other health conditions

**Glaucoma:**

- Eyes with long-standing glaucoma have poor endothelial cell counts; postoperative corneal oedema may occur.
- Eyes that have been treated for many years with anti-glaucoma agents, like pilocarpine, may have pupils resistant to dilation.
- Complications like iris injury, capsular tear and zonular dialysis can all aggravate pre-existing glaucoma.
- Previous trabeculectomy means that the functioning bleb must be preserved during cataract surgery, by using either a corneal incision (phacoemulsification) or a temporal approach. To complicate matters, synechiae and a shallow anterior chamber are often present.

**HIV-positive patients:** cataract surgery in these patients requires routine (and thus proper) care. However, such patients may have posterior segment complications such as cytomegalovirus (CMV) retinitis, vasculitis, and choroiditis, which may not be evident in a white cataract. Performing a B-scan may not always be helpful, but it should be done when fundus details are not clear. These patients are also prone to secondary infection.

**Diabetes:** it is important to try and keep the posterior capsule intact. Retinopathy progresses more rapidly in diabetic patients after cataract surgery and a ruptured capsule can be a factor in rubeosis. Close follow-up and timely laser treatment are required. If possible, treat the retinopathy preoperatively with laser.

**Onchocerciasis:** this disease affects the cornea, uvea, and retina. In endemic areas, cataract surgery can be disappointing due to optic nerve and retinal pathology. You must take care when selecting patients for cataract surgery, in order to avoid performing operations which will bring no benefit to patients.

**Hypertension and high positive pressure during surgery:** in general, it is important to avoid a high positive pressure during surgery. This can be caused by an inadequate or excessive peribulbar block, or a tight bridle suture. It is therefore important to control hypertension in patients. In addition, retrobulbar haemorrhage should be identified early and the operation postponed. Expulsive haemorrhage in one eye could alert you to possible problems in the second eye.

**Asthma, chronic obstructive pulmonary disease and constipation**: when in doubt about whether to suture the wound, it is always better to do so – especially in patients suffering from these conditions.
